# A model to estimate seabird field metabolic rates

**DOI:** 10.1098/rsbl.2018.0190

**Published:** 2018-06-06

**Authors:** Ruth E. Dunn, Craig R. White, Jonathan A. Green

**Affiliations:** 1 School of Environmental Sciences, University of Liverpool, Liverpool L69 3GP, UK; 2 Centre for Geometric Biology, School of Biological Sciences, Monash University, Melbourne, Victoria 3800, Australia

**Keywords:** breeding season, energetics, energy expenditure, field metabolic rate, meta-analysis, seabirds

## Abstract

For free-ranging animals, field metabolic rate (FMR) is the sum of their energy expenditure over a specified period. This quantity is a key component of ecological processes at every biological level. We applied a phylogenetically informed meta-analytical approach to identify the large-scale determinants of FMR in seabirds during the breeding season. Using data from 64 studies of energetics in 47 species, we created a model to estimate FMR for any seabird population. We found that FMR was positively influenced by body mass and colony latitude and that it increased throughout the breeding season from incubation to brood to crèche. FMR was not impacted by colony-relative predation pressure or species average brood size. Based on this model, we present an app through which users can generate estimates of FMR for any population of breeding seabird. We encourage the use of this app to complement behavioural studies and increase understanding of how energetic demands influence the role of seabirds as driving components of marine systems.

## 1. Introduction

Metabolic energy requirements drive biological processes at every hierarchical level of life. At the organismal level, field metabolic rate (FMR) is the total sum of energy that a free-ranging animal metabolizes over a specified period of time. Understanding the determinants of interspecific FMR helps us to quantify the impact that free-ranging animals have on energy flows within the ecosystems that they inhabit [1].

It has long been known that body size is a key determinant of FMR between organisms of the same taxonomic class, accounting for around 95% of within-class variation [1]. However, the magnitude of the remaining interspecific variation in metabolic rate can be considerable and is determined by a number of other physiological and ecological factors. For example, latitude (which encompasses variation in air temperature, sea surface temperature, productivity, day length and seasonality) positively influences FMR in small mammals owing to cooler habitat temperatures and consequent increased thermoregulatory energetic costs [2]. Similarly, while energetic bottlenecks may occur at different points throughout the annual cycle, birds often exert high metabolic rates during the reproductive season owing to the increased energetic costs associated with egg incubation and offspring provision [3–5]. More recently, additional factors such as colony size and number of offspring have been suggested as drivers of FMR within free-ranging animals such as colonially breeding seabirds [6,7].

Studies on the metabolic rates of seabird species have increased dramatically in recent decades [7]. This is due to both their tractability and the need to better understand the food requirements of this important yet threatened group [8]. To date, the majority of studies have focused on the energetically demanding reproductive period when seabirds are constrained to travel potentially large distances between the breeding colony and marine feeding areas [9]. Despite the need to understand the metabolic requirements of marine top-predators for conservation purposes, the FMR of many seabird species and populations remains unknown and the broad-scale determinants of interspecific variation in seabird FMR are unclear.

Here, we applied a phylogenetically informed meta-analytical approach to explore the large-scale determinants of seabird FMR during the breeding season, updating previous studies on the correlates of seabird FMR [7]. In addition, we present this model within a web-based app that can be used to make estimates of FMR for seabird species and populations where this has not previously been calculated.

## 2. Material and methods

### (a) Data compilation

A systematic search of the peer-reviewed literature was conducted between November 2016 and January 2018 inclusive, including all records until this time. We used a combination of the following keywords: ‘seabird*', ‘energ*', ‘field metabol*' and ‘rate' to search the Web of Science and Google Scholar. Abstracts were scanned for an indication that publications reported measurements of energy expenditure and where appropriate the full text was then consulted.

Values of FMR (*n* = 98), calculated using doubly labelled water or heart rate loggers or via the construction of time–energy budgets, were obtained from 64 original studies on 47 species of seabird. Additionally, values of mean bird mass, phase of breeding season (incubation, brood or crèche), colony name, latitude and number of breeding pairs at the colony were recorded. When these data were not available within the original studies, we contacted the authors or consulted further literature to obtain them.

### (b) Statistical analysis

Phylogenetic meta-analytic models to identify the large-scale determinants of seabird FMR and to make predictions of FMR were constructed in the R environment [10] using the MCMCglmm package [11]. Models included combinations of the following fixed effects: log-transformed mean bird mass, species average brood size, phase of breeding season, colony latitude and colony-relative predation pressure (the log-transformed product of the number of breeding pairs and bird mass^2/3^). We accounted for the potential non-independence of data due to shared ancestry by including a phylogenetic random effect alongside species and colony. To incorporate phylogeny we used the Ericson backbone tree downloaded from http://birdtree.org/ [12]. The tree was pruned to only include 313 seabird species (see electronic supplementary material, S1). Log-transformed FMR was modelled as a Gaussian response variable and parameter-expanding priors were used for the random effects. The MCMC chains were run for a total of 260 000 iterations with a burn-in of 60 000 and thinning interval of 200. The best model (that which incorporated the optimum combination of fixed effects) was selected using the deviance information criteria (DIC) [13]. Graphic diagnostics were used to assess for autocorrelation, and jackknife analysis was used to resample the data and check the resulting model (see electronic supplementary material, figure S2). An estimate of phylogenetic heritability (*H^2^*) was calculated to provide an index of the proportion of variance associated with the random effect of phylogeny [14].

## 3. Results

**Figure 1.**
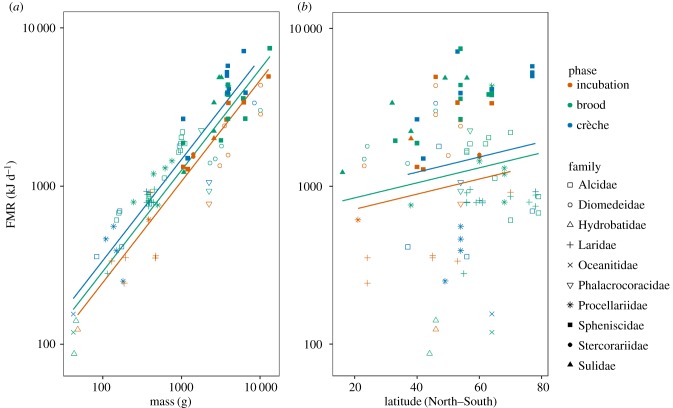
Breeding seabird field metabolic rate (FMR) was modelled as a function of (*a*) bird mass and (*b*) latitude. The colours of the points and model fit lines represent the stage of the breeding season, and the shape of the points corresponds with the family. Mass and FMR axes are displayed as a logarithmic scale.

All models were within two DIC values and were, therefore, considered to provide comparably good fits to the data (see electronic supplementary material, table S3). All models showed similar positive effects of bird mass and absolute latitude on FMR in breeding seabirds (figure 1), with phase of the breeding season also having an impact. Conversely, models did not provide strong evidence to support that species average brood size or colony-relative predation pressure impacted FMR, and the phylogenetic heritability was low (see electronic supplementary material, table S3).

While all models were competitive and suggested similar results, the simplest model with the lowest DIC was considered the strongest (table 1). This model was incorporated within the R shiny web framework [15] to create a web-based utility and user interface through which to generate estimates of seabird FMR. The app requires inputs of species, bird mass, colony latitude and phase of breeding and returns a daily FMR estimate alongside HPD confidence intervals, based on the optimal model. The ‘Seabird FMR Calculator’ web app is available at https://ruthedunn.shinyapps.io/seabird_fmr_calculator/.

**Table 1.** Results from the random-effects meta-analyses on the large-scale drivers of seabird field metabolic rate during the breeding season.

effect	posterior estimates	lower 95% CI	upper 95% CI	*p*_MCMC_
intercept (brood)	0.92	0.62	1.21	<0.001
breeding phase: incubation	−0.071	−0.12	−0.025	0.002
breeding phase: crèche	0.068	0.027	0.11	0.006
log bird mass	0.64	0.55	0.72	<0.001
colony latitude	0.0048	0.0023	0.0073	0.002
*H^2^* heritability estimate	mean = 0.035; s.d. = 0.019			

## 4. Discussion

This study uses the most comprehensive methods available to provide the best and most up-to-date analyses of the large-scale determinants of seabird FMR during the breeding season. The results of our phylogenetically informed meta-analyses indicate a lack of evidence of a phylogenetic signal and, therefore, suggest that mean bird mass, absolute latitude and phase of the breeding period are more influential predictors of FMR in breeding seabirds than phylogeny.

We observed an increase in FMR across the breeding season from incubation to brood to crèche (figure 1). Although incubation can be an energetically costly period for seabirds, owing to its intrinsic costs and those of its associated activities [4,16], some species-specific studies have shown increased FMR later in the breeding season owing to elevated basal metabolic rates and the energetic costs associated with offspring provision [5,17]. Our findings support these previous studies of energy expenditure in individual populations of seabird and extend them to identify a link between FMR and phase of the breeding period across a range of seabird species.

While an organism's mass is well known to influence its energy expenditure, geographical relationships have been less frequently explored across such a breadth of taxa. Our study supports the hypothesis that in response to adverse environmental conditions, seabirds breeding at high latitudes have higher FMR (figure 1). These increased rates of energy expenditure may be due to elevated metabolism and adjustments to metabolic rhythms in response to cooler temperatures, longer days, shorter breeding seasons and other climatic effects associated with high latitudes [3,18,19].

It has been proposed that seabird colonies may be surrounded by a ‘halo' of depleted prey availability during the breeding season owing to increased feeding activities in the vicinity of the breeding colony [20,21]. Local prey depletion might be greatest around large colonies, and this might require individuals at larger colonies to travel greater distances to forage [22]. While Adélie penguins nesting in larger colonies, therefore, travel further to access prey resources, expending more energy in order to do so [6], our cross-species analyses did not find general support for this hypothesis. Instead, we found that neither colony-relative predation pressure nor species average brood size influenced estimates of breeding seabird FMR. This lack of a distinguishable relationship may be due to the fact that the ‘halo' argument has previously only been validated regionally, whereas our analyses include data that encompass a vast range of marine habitats and consequentially a high variance of prey availability. Furthermore, while brood size might influence intraspecific FMR [23,24], at the species-level FMR is set by life-history trade-offs for which the animal will have reallocated its energetic resources [25]. Alternatively, our results might suggest a common optimal rate of FMR across taxonomic groups [26], given the internal demands of chick rearing and the external influence of latitude.

We use our model to present a user-friendly web-based app (the ‘Seabird FMR Calculator'). This app uses data on bird mass, colony latitude and phase of the breeding period, to calculate estimates and confidence intervals of FMR for any seabird population. Such estimates of FMR are essential when inferring the food consumption of populations of seabirds across multiple temporal scales [8] and also when parametrizing mechanistic models to make energetic predictions in a climate change context (e.g. [27]). We, therefore, envisage that outputs from the ‘Seabird FMR Calculator' can be encompassed within future studies in order to increase understanding of the energetic demands of these top predators, their role within the wider marine ecosystem and how this might be influenced by climatic change. The creation of this app is particularly timely owing to the competition pressures that seabirds, key driving components of marine systems, face from anthropogenic activities such as the depletion of marine stocks by global fisheries [28]. The conservation of seabird populations is, therefore, of vital importance and we encourage that the ‘Seabird FMR Calculator' is used as a key tool at the forefront of these efforts. In addition, we advocate the ‘Seabird FMR Calculator' as a prototype for the development of similar apps that, in turn, can be used to make estimations of FMR for a wider range of taxa for which this information is available (e.g. marine mammals, marsupials, passerines and lizards [29]).

## Supplementary Material

S1. Seabird species selection process; Figure S2. FMR values and jackknife estimates; Table S3. Model outputs.

## Data accessibility

Meta-analysis data and phylogeny used are publicly hosted on figshare. The data are accessible at http://dx.doi.org/10.6084/m9.figshare.4765906 [30] and the phylogeny is accessible at http://dx.doi.org/10.6084/m9.figshare.5972692 [31].
