# Supplementary material for: A model to estimate seabird field metabolic rates
Source: Biol Lett. 2018 Jun 6;14(6):20180190. doi: 10.1098/rsbl.2018.0190 (PMC6030596; doi:10.1098/rsbl.2018.0190)
Supplement: S1. Seabird species selection process; Figure S2. FMR values and jackknife estimates; Table S3. Model outputs. [file rsbl20180190supp1.docx]

**Electronic Supplementary Material**

S1.

The 346 seabird species, as defined by Croxall et al. (2012), were initially selected for inclusion within the phylogenetically-controlled analyses. Species within the Anatidae (n = 18), Gaviidae (n = 5), Podicipediformes (n = 4) and Scolopacidae (n = 2) families were then removed since the life-history strategies of these species are different to those typical of the other seabird families. In addition, 4 extinct seabird species (Olson’s petrel, *Bulweria bifax;* Guadalupe storm petrel, *Oceanodroma macrodactyla;* Great auk, *Pinguinus impennis* and Saint Helena petrel, *Pterodroma rupinarum*) were also removed*.*


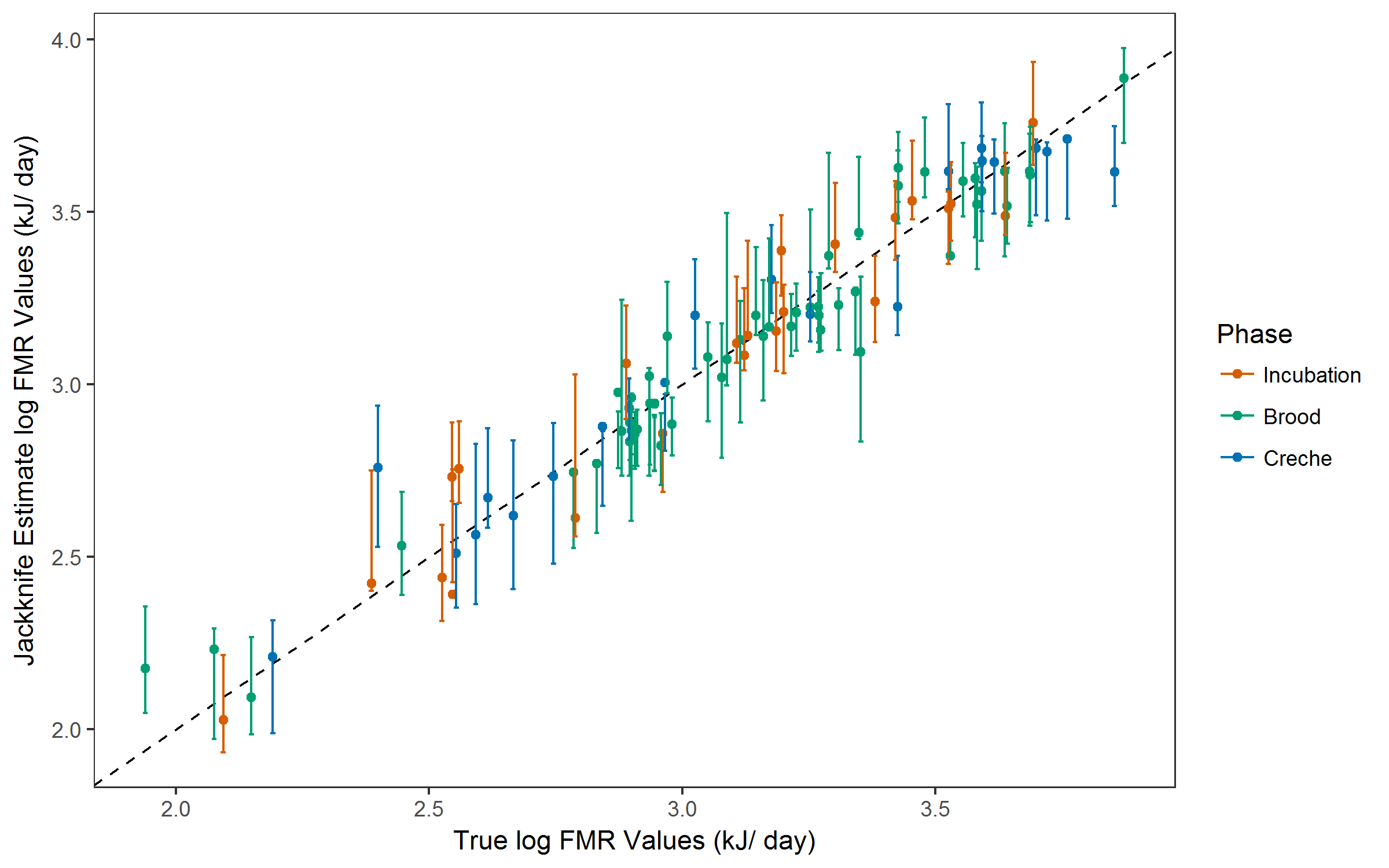


Figure S2. Values of FMR extracted from the literature and posterior estimates of FMR produced by jackknife analyses. Error bars represent lower and upper 95% credible intervals.

When jackknife estimates were plotted against the original values upon which the model was built, a linear relationship was detected, with no evidence of systematic bias.

Table S3. DIC values, variance components (species, phylogeny, colony and residual) and phylogenetic heritability (*H^2^*; mean ± standard deviation) for 4 models comparing the key drivers of field metabolic rate (FMR) in breeding seabirds. Asterixis indicate pMCMC < 0.005. The optimum model is highlighted in bold.

| Fixed effects | DIC | Variance components | | | | *H^2^* |
| --- | --- | --- | --- | --- | --- | --- |
|  |  | Species | Colony | Phylogeny | Residual |  |
| Mass*; latitude*; breeding stage*; brood size; pairs × mass^2/3^ | -217.80 | 0.0025 | 0.0034 | 0.00032 | 0.0039 | 0.034 ± 0.017 |
| Mass*; latitude*; breeding stage*; pairs × mass^2/3^ | -218.65 | 0.0025 | 0.0035 | 0.00033 | 0.0039 | 0.035 ± 0.018 |
| Mass*; latitude*; breeding stage*; brood size | -218.42 | 0.0025 | 0.0033 | 0.00032 | 0.0040 | 0.034 ± 0.020 |
| **Mass*; latitude*; breeding stage*** | **-219.50** | **0.0025** | **0.0035** | **0.00033** | **0.0039** | **0.035 ± 0.019** |
